# Action recommendations review in community-based therapy and depression and anxiety outcomes: a machine learning approach

**DOI:** 10.1186/s12888-024-05570-0

**Published:** 2024-02-16

**Authors:** Amit Spinrad, C. Barr Taylor, Josef I. Ruzek, Samuel Jefroykin, Tamar Friedlander, Israela Feleke, Hila Lev-Ari, Natalia Szapiro, Shiri Sadeh-Sharvit

**Affiliations:** 1 Eleos Health, 117 Kendrick Street, Suite 300, Needham, MA 02494 USA; 2 Center for m2Health, Palo Alto University, Palo Alto, CA USA; 3 Department of Psychiatry, Stanford Medical Center, Stanford, CA USA

**Keywords:** Activation, Deep learning, Empirically-based practice, Natural language processing, Behavioral treatment, Machine learning, Homework

## Abstract

**Background:**

While the positive impact of homework completion on symptom alleviation is well-established, the pivotal role of therapists in reviewing these assignments has been under-investigated. This study examined therapists' practice of assigning and reviewing action recommendations in therapy sessions, and how it correlates with patients’ depression and anxiety outcomes.

**Methods:**

We analyzed 2,444 therapy sessions from community-based behavioral health programs. Machine learning models and natural language processing techniques were deployed to discern action recommendations and their subsequent reviews. The extent of the review was quantified by measuring the proportion of session dialogues reviewing action recommendations, a metric we refer to as “review percentage”. Using Generalized Estimating Equations modeling, we evaluated the correlation between this metric and changes in clients' depression and anxiety scores.

**Results:**

Our models achieved 76% precision in capturing action recommendations and 71.1% in reviewing them. Using these models, we found that therapists typically provided clients with one to eight action recommendations per session to engage in outside therapy. However, only half of the sessions included a review of previously assigned action recommendations. We identified a significant interaction between the initial depression score and the review percentage (*p* = 0.045). When adjusting for this relationship, the review percentage was positively and significantly associated with a reduction in depression score (*p* = 0.032). This suggests that more frequent review of action recommendations in therapy relates to greater improvement in depression symptoms. Further analyses highlighted this association for mild depression (*p* = 0.024), but not for anxiety or moderate to severe depression.

**Conclusions:**

An observed positive association exists between therapists’ review of previous sessions’ action recommendations and improved treatment outcomes among clients with mild depression, highlighting the possible advantages of consistently revisiting therapeutic homework in real-world therapy settings. Results underscore the importance of developing effective strategies to help therapists maintain continuity between therapy sessions, potentially enhancing the impact of therapy.

## Introduction

In the current mental health crisis, identifying the most effective ingredients of therapy is of utmost importance, as there is a scarcity of trained mental health providers [1]. Furthermore, the emotional, financial, and societal costs of ineffective therapy are significant concerns [2]. Ineffective therapy can lead to prolonged suffering, limited improvement in symptoms, and hindered functioning for individuals seeking help. This not only affects their well-being, but also creates a strain on resources [3]. Therefore, it is imperative to investigate which specific aspects of therapy are most strongly associated with symptom reduction, so that treatment can be optimized, and resources can be allocated most efficiently. By identifying the ingredients of therapy that are most effective, we can improve the quality of mental health care and help alleviate the burden of mental illness on individuals, families, and society as a whole [4].

The mechanisms influencing the effectiveness of therapeutic interventions encompass a diverse array of factors. Robust treatment outcomes are closely tied to the strength of the therapeutic alliance [5], the caliber of specialized training, and the consistent supervision of therapists in delivering empirically-supported interventions [6]. Among these interventions, therapeutic homework stands out as one of the most extensively studied techniques in the realm of therapy for depressive and anxiety disorders [7]. The assignment of therapeutic homework, a practice integral to time-limited interventions, has consistently demonstrated its capacity to predict treatment outcomes. While existing literature underscores the broad impact of various factors on treatment efficacy, this study delves into the nuanced exploration of a specific aspect—the review of action recommendations within the context of homework dynamics—aiming to contribute valuable insights to this multifaceted landscape, especially concerning its implications for the treatment of depression and anxiety [8].

Compliance with assigned homework predicts improved outcomes for various conditions, such as anxiety, depression, and substance use [8]. A recent study found that therapists assigned homework in 61% of sessions carried out in real-world practice settings [9]. However, little is known about whether these homework assignments are reviewed in subsequent sessions. One form of homework that may be particularly effective in time-limited therapy is behavioral activation (i.e., encouraging clients to engage in more behaviors intended to increase pleasure and reduce suffering between sessions). It has been found that behavioral activation is a predominant ingredient in various treatment modalities for depression and anxiety [10–12]. Behavioral activation can be also implemented as a distinct treatment intervention that includes structured homework assignments, where clients are given specific tasks and activities designed to target specific behaviors related to pleasure and meaningful engagement. However, it is important to note that behavioral activation is not limited to one treatment approach; it can also be integrated into other therapeutic modalities as part of the homework assignments [13]. Therapists can incorporate behavioral activation strategies and encourage clients to engage in behaviors that align with their treatment goals, regardless of the specific therapeutic approach being used. This flexibility allows for individualized treatment planning and the integration of behavioral activation within the broader framework of therapy homework, which can include a range of therapeutic techniques and interventions tailored to each client's unique needs and preferences [14].

Studies have focused on client variables in homework adherence and compliance, indicating that greater symptom reduction is found in clients completing at least half of assigned homework [15]. However, the therapist’s contribution to homework compliance has remained understudied. Achieving high levels of client homework compliance is contingent upon the therapist's ability to carefully select, plan, and review homework assignments, with the aim of optimizing the clinical utility and practical feasibility of the homework [16]. Despite ongoing efforts to promote evidence-based practices (EBPs) in behavioral health settings, widespread implementation of EBPs remains a challenge [17]. Studies have shown that therapists' adoption of EBPs in routine practice is still relatively low, which can hinder the potential benefits of these practices for clients [18, 19]. While therapy homework is considered one of the key components of EBPs and has demonstrated effectiveness in various interventions, its standardized implementation can be challenging in real-world settings [20]. To address this issue, this study focused on exploring all activation recommendations made by therapists in therapy sessions, rather than solely focusing on standardized homework assignments. Examining a broader range of activation recommendations can capture a more comprehensive view of the interventions and strategies therapists use to extend the impact of therapy. By reviewing and revisiting activation recommendations in subsequent sessions, therapists can refine their homework assignments and help clients adhere to homework assignments, maintain progress, and continue to engage in adaptive behaviors.

Until recently, understanding the processes underlying effective therapy has relied on self-reported data from therapists or audio recordings of sessions collected in research settings, both of which have limitations in terms of potential bias and generalizability [21]. Furthermore, it remains unclear whether therapists actively review homework assignments with clients in routine care and whether this practice is related to better therapeutic outcomes. To address these gaps in the literature, the aim of this study is to examine the relationship between reviewing actions recommended by the therapist and treatment outcomes for depression and anxiety. By collecting data on therapist review of assigned activities in a naturalistic setting, we explore potential correlations that might offer new insights into the mechanisms underlying effective therapy. Findings from this study could contribute to the body of knowledge that informs therapist training and suggests avenues for improving therapeutic outcomes, ultimately benefiting patients, and addressing the mental health crisis by helping maximize the effectiveness of therapy.

## Methods

### Settings and interventions

This study involved examining fully anonymized data from behavioral treatments in 14 behavioral programs across the United States. The study included clients who received individual therapy in either outpatient or intensive outpatient programs for various mental health issues. The therapists were licensed psychologists, social workers, or counselors providing time-limited therapy for various mental health concerns. Sessions were processed through the Eleos Health proprietary Artificial Intelligence (AI) platform [22]. We selected clients who completed the Patient Health Questionnaire-9 (PHQ-9) [23] and the Generalized Anxiety Disorder-7 (GAD-7) [24] assessments twice, with the two evaluations being 50–70 days apart. Within this time period, clients were required to attend a minimum of three different therapy sessions, at least 5 days apart. To be included in the analysis, clients were also required to have a minimum initial score of 5 on either assessment, indicating at least mild depression or anxiety. Furthermore, the duration of sessions for the analysis was capped at a range of 15 to 90 min. The final dataset comprised a unique total of 450 clients treated by 126 therapists in 2,444 therapy sessions. Within this sample, 398 clients and their corresponding sessions were included in the PHQ-9 dataset, while 412 clients were part of the GAD-7 dataset, as not all 450 clients had both assessments. Demographic data (age and gender) were available for a subset of approximately 200 clients. We chose not to perform data imputation due to the large amount of missing demographic data, which could potentially introduce bias into the results. This study was approved by Sterling IRB external institutional research board, #9545, and informed consent was obtained from all clients. Both therapists and clients had the option to opt out and not use the Eleos platform during their sessions.

### Initial data analyses

To accurately analyze speech data from behavioral treatment sessions and identify therapeutic strategies, we processed the data to transcribe sessions, identify the therapist and the client, and label the topics discussed in the session [9]. All sessions were fully transcribed using automatic speech recognition and a domain-specific text-cleaning algorithm, achieving a 98% accuracy rate in distinguishing between speakers in therapy sessions at the session level [21]. Conversations were subsequently divided into micro-dialogues, each comprising approximately 300 words. Each micro-dialogue included both therapist and client utterances and revolved around a specific topic [25]. If a session contained ten or fewer micro-dialogues, or lasted less than 15 min, it and the subsequent session were excluded from the data.

### Models development

An initial review of the session data indicated that therapists tend to recommend activities that the client will engage in between the sessions, and that these homework assignments are frequently action recommendations phrased as general advice (e.g., “Why don’t you practice some cooking this week to see whether this affects your eating habits?”; “So we agree that you will schedule an appointment with your doctor to go over medications”; and “I think it would be great if we could work on finding ways to address your communication problems with your partner”). Therefore, “action recommendation” was defined as an activity recommended by the therapist that is in line with the treatment goals, but not assigned a specific day and time and is not necessarily expected to be completed by the next session. We use the term “action recommendations” to differentiate it from the narrower focus of behavioral activation.

The unstructured texts from client-therapist interactions were initially processed by 5 experts, all of whom were graduate-level clinical psychologists or social workers with a minimum of two years of experience in providing therapy. These experts were responsible for both annotating and summarizing the content throughout the tasks mentioned hereinafter.

We developed two language models to facilitate the extraction of action recommendations from each session. First, we fine-tuned a pre-trained Bidirectional Auto-Regressive Transformers (BART) [26] model to classify the therapist-client micro-dialogues that discussed future plans (e.g., discussing a public speaking event planned for next week). This was done using a training dataset consisting of preliminary classifications of 1,191 micro-dialogues from multiple therapy sessions, evaluated and labeled by 3 experts, as either indicative or not indicative of future plans. Second, we trained a Passive Aggressive Classifier [27] primarily employing term frequency-inverse document frequency (TF-IDF) features, using 311 micro-dialogues classified by 3 experts, to ascertain whether micro-dialogues which were classified as “plans” qualified as “action recommendations.” This was achieved using a similarly compiled training dataset labeled by the same experts.

Next, we developed two generative AI models, both based on pre-trained BART models and fine-tuned using datasets containing summaries of relevant micro-dialogues. These summaries were created by the 5 experts mentioned above for two distinct tasks: specific action recommendations-related summary (using 179 summaries) and “general” dialogue content summary (using 642 summaries). The fine-tuning of these models utilized these experts’ summaries. Following this step, the first model demonstrated an ability to generate summaries of micro-dialogues that encompass action recommendations within a specific session. The second model generated more “general content” summaries of all micro-dialogues in a given session, irrespective of their classification.

Finally, we developed an “action recommendations review” algorithm, which utilized a sentence transformers model [28] trained on 215 million question-and-answer pairs (multi-qa-mpnet-base-dot-v1) to perform a semantic search. Using this approach, we calculated semantic similarity between pairs consisting of a general micro-dialogue summary of a session and the previous session's action recommendation summary. Summaries of general micro-dialogues—exceeding a set similarity threshold in comparison to the previous session's action recommendation summary—were deemed as reviewing the specific action recommendation. This threshold was established based on the optimal F-score from a dataset of 90 such pairs, which were labeled by two experts of the 5 abovementioned.

To test inter-rater reliability, we calculated both the percentage of agreement between the experts as well as Cohen's Kappa [29, 30]. These assessments were conducted using two distinct classification datasets, containing 220 data points.

### Model validation

After fine-tuning and training our models and algorithms, and then testing them on the remaining 15–30% of the training data initially reserved for validation, we further validated them using independent datasets excluded from the training process. This included:

1. Validating the action recommendations classification model with two independent datasets: one featuring full transcripts evaluated by experts, and another featuring 80 micro-dialogues from various sessions, classified by our models as containing action recommendations and verified by experts.
2. Validating the action recommendations summary model and the general micro-dialogues summarization model. The former was evaluated using correctly-classified action recommendations from the dataset mentioned in (1), where evaluators assessed the accuracy of the summaries, using a 3-categories scale: “mostly true”, “partly true” and “mostly false”. The latter was tested using a dataset of 100 general content micro-dialogues and their corresponding automatic summaries, assessed using the same methodology.
3. Validating the action recommendation review algorithm using a dataset of 200 pairs of session and previous session summaries. Evaluators, blind to the set similarity score and threshold, assessed whether the general micro-dialogues’ summaries genuinely reviewed their corresponding action recommendation summaries.

In these validation processes, “precision” and “recall” were used to measure the classification models' efficacy. Precision assessed the accuracy of our models when classifying a dialogue as incorporating an action recommendation or a review, while recall measured our models' ability to correctly identify all relevant instances.

### Final statistical analyses

We investigated the association of therapists’ review of prior action recommendations with clients' symptom improvement by calculating, for each session (beginning from the second one for each client), the proportion of micro-dialogues that referred to recommendations from the previous session (a metric we termed "review percentage"). Change in depression and anxiety scores (i.e., clients’ symptom change) was calculated using the formula $( \frac{initial score-final score}{initial score} )*100$). In this calculation, a positive value indicates symptom improvement, and a negative value indicates symptom deterioration, consistent with established practices in clinical research [23, 31]. For instance, a change score of 50% suggests a reduction in symptom severity (e.g., a PHQ-9 score change from 8 to 4).

For visualization purposes, as illustrated later in Fig. 2, we standardized the symptom change scores to range between -100% to 100%. This was intended to facilitate a clear visual representation of the results and was not used in any statistical modeling process. Therefore, the standardized change scores should be interpreted as consistent with the original change scores, with positive values indicating symptom improvement and negative values indicating symptom deterioration.

The association of this "review percentage" with changes in depression and anxiety scores was examined using Generalized Estimating Equations (GEE) modeling. This approach was chosen due to its capacity to handle the nested structure of our data (multiple sessions within clients) [32] and its less strict assumptions regarding the outcome variable distribution and variance across clusters [33]. Moreover, one of the advantages of GEE is its focus on estimating population-average effects rather than the variability within clusters, which aligns with this study’s overarching research question.

In the GEE analyses, we controlled for factors that can potentially introduce bias like the behavioral health organization, therapist, number of micro-dialogues per session, number of prior session's action recommendations, initial symptom score, and assessment recording duration (in days). To avoid unnecessary complexity in the presentation of the results, the effects of therapists and organizations were not detailed in later post-hoc exploratory models (as presented in Tables 3 and 4).

To ensure the robustness of our analysis, we applied transformations on the primary variables to handle potential data skewness and outliers [34]. Specifically, we chose a cube-root transformation for the symptom score change variable, which contained a mix of negative, zero, and positive values. Conversely, for the review percentage variable, which only included zero and positive values, we employed a log transformation. This transformation, computing the natural logarithm of 1 + x for all x in the input, was a more straightforward choice and was feasible due to the inherent absence of negative review percentages. Importantly, this transformation approach accommodated zero values, thereby avoiding undefined results [34].

We further validated our findings by using a non-parametric bootstrap method with 1,000 samples, which served as an additional check on the significance of our results [35].

Whenever a significant association was not identified in the initial GEE modeling, we conducted further examination of the data, focusing on patterns and associations among the other covariates, as well as potential interactions. We then conducted subsequent post-hoc observational analyses, including GEE analyses for each client’s baseline category of depression (PHQ-9) and anxiety (GAD-7) separately, without interaction terms.

**Fig. 1:**
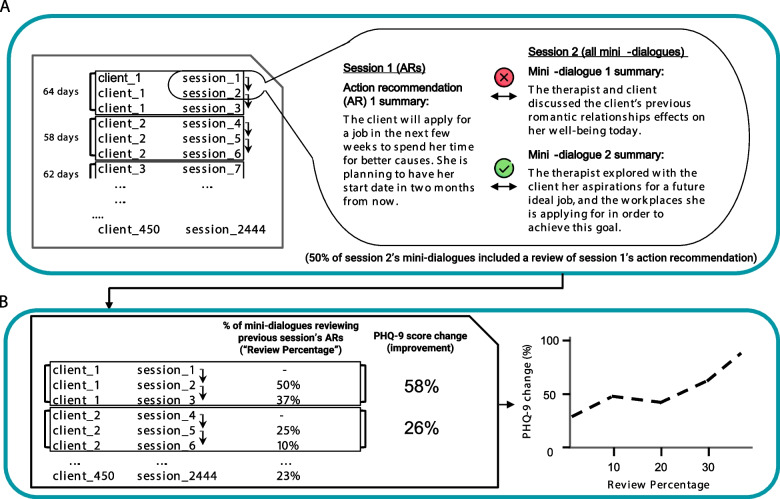
The study’s analytic pipeline. **A** (Left) The primary dataset analyzed, consisting of 450 clients and 2,444 therapy sessions. For each client, we extracted the number of days between two assessments. (Right) For each pair of consecutive sessions, we assessed whether action recommendations given in the former session were reviewed in the latter session’s dialogues and calculated the number of these occurrences & their percentage. **B** (Left) Using these calculations, we generated a comprehensive table containing all the data from (**A**) along with the main calculated metric for each session: the “Review Percentage”. (Right) With the complete data in hand, we examined the relationship between the review percentage and the changes in both depression (PHQ-9) and anxiety (GAD-7) during treatment (here, an illustration of PHQ-9 is shown; this figure is an illustrative representation of our findings, rather than a direct display of the actual results)

The statistical analyses were performed using Python’s statsmodels package v0.13.5 [36] and scikit-learn package v0.24.2 [37]. Figure 1 provides a simplified visual description of the analytic process.

## Results

### Sample characteristics

Table 1 shows the characteristics of the final analyzed sample, with distinct analyses for both PHQ-9 and GAD-7. The size of the analyzed sample is marginally smaller than the initially stated sample size, as indicated in the methods section. This difference is due to our focus on data from each client's second session onward, to ensure that we had the required action recommendations from the previous session for our analysis. Of note, the majority of clients had both GAD-7 and PHQ-9 assessments recorded within the examined data. As a result, the datasets for each assessment largely overlap in terms of client population, leading to identical or near-identical values for certain variables across both datasets. These variables are the number of sessions analyzed per client, the number of micro-dialogues per session, the number of action recommendations per session, and the analysis timeframe.

**Table 1:** Sample characteristics

**Variable**	**GAD-7** [Mean (Standard Deviation)]	**PHQ-9** [Mean (Standard Deviation)]
N (clients)	412	398
N (sessions)	1,757	1,839
Gender (Female, percent)^a^	70.4%	70.2%
Age^a^	37.1 (16.3)	36.6 (16.2)
Initial score	11.1 (4.8)	12.2 (5.7)
Final score	7.2 (5.3)	8.4 (6.5)
Number of sessions per client	5.2 (2.1)	5.2 (2.1)
Change in score (percent)	30.7% (52.2)	28.3% (53.8)
Change in score, standardized (percent)	60.4% (29.8)	57.8% (31.7)
Number of micro-dialogues per session	31.5 (7.7)	31.3 (7.8)
Number of action recommendations per session	4.7 (3.2)	4.7 (3.2)
Analysis time-frame (days)	60.8 (4.6)	60.8 (4.5)

^a^denotes partial data; demographic data (age and gender) were available for 186 and 178 clients in the GAD-7 and PHQ-9 datasets (45.1% and 44.7%), respectively

### Inter-rater reliability

Inter-rater agreement on the classification datasets, involving 4 experts, ranged from 89.4% for action recommendation review classification, to 93.3%, for action recommendation classification. Cohen's Kappa scores were 0.63 for action recommendation classification and 0.75 for action recommendation review classification, indicating a substantial level of agreement [29].

### Action recommendation classification evaluation

Our classification approach, which utilized two sequential classification models, successfully identified micro-dialogues containing action recommendations with an overall precision of 76% and a recall rate of 71.4%.

### Summarization evaluation

In our evaluation of the general and action recommendation summary models, we found that 92.4% and 78.6% of micro-dialogue summaries, respectively, were labeled by expert reviewers as "mostly true" or “partly true” in adequately representing the dialogue content.

### Review of action recommendation evaluation

Our method for classifying whether a micro-dialogue includes a review of an action recommendation achieved a precision of 71.1% and a recall rate of 72.7%.

### Overview of session data

Our models indicated that in both the PHQ-9 and GAD-7 datasets, 94.9% and 95% of the sessions, respectively, included at least one micro-dialogue containing an action recommendation. However, approximately half of these sessions incorporated micro-dialogues reviewing action recommendations from previous sessions—specifically, 49.5% in the PHQ-9 dataset and 50% in the GAD-7 dataset.

### Generalized Estimating Equation regression analysis

Multivariate GEE regression analyses were carried out on both the PHQ-9 and GAD-7 datasets as described in the methods section, with the results summarized in Table 2. The examination of these results revealed that, for both datasets, there was no significant association between the review percentage and the change in depression and anxiety after adjusting for other variables (*p* = 0.846 and *p* = 0.659 for the GAD-7 and PHQ-9 datasets, respectively, Table 2).

**Table 2:** Results from Generalized Estimating Equations modeling for the depression and anxiety datasets

**Variable**	PHQ9		GAD7	
	**Coefficient (*p*-value)**	**Coefficient CI [0.025 0.975]**	**Coefficient (*p*-value)**	**Coefficient CI [0.025 0.975]**
Intercept	-1.32 (0.561)	[-5.79, 3.14]	-1.77 (0.319)	[-5.25, 1.71]
**Log (Review Percentage)**	0.03 (0.659)	[-0.11, 0.17]	-0.01 (0.846)	[-0.14, 0.12]
Number of Dialogues/Session	0.03 (0.029*)	[0.00, 0.06]	0.01 (0.384)	[-0.01, 0.04]
Number of Action Recommendations/Previous Session	0.01 (0.819)	[-0.06, 0.07]	0.03 (0.408)	[-0.04, 0.09]
Client's Initial Score	0.06 (0.023*)	[0.01, 0.12]	0.13 (0.000*)	[0.08, 0.19]
Days Examined	0.02 (569)	[-0.06, 0.10]	0.02 (0.518)	[-0.04, 0.08]
Therapist Fixed Effects	–**	-	–**	-
Organization Fixed Effects	–**	-	–**	-

^*^denotes *p* < 0.05. **There were 67 & 72 significant therapist fixed effects and 7 & 9 significant organization fixed effects found in the GEE models of PHQ-9 and GAD-7, respectively. Variables transformed: review percentage logged, score change cube-rooted (the dependent variable). All significant associations were verified via bootstrap resampling (1000 samples)

In both datasets and analyses, the initial anxiety or depression score was found to have a significant correlation with the change in score. This led to further exploration into the potential interaction of the initial anxiety/depression score with the review percentage variable, in relation to score change. The interaction was found to be significant, but only within the PHQ-9 dataset (*p* = 0.045, Table 3). However, when this interaction was added to the analysis and thus was controlled, the review percentage variable association with the change in depression score was found as significant as well (*p* = 0.032, Table 3). The interaction term between the review variable and the change in depression score carried a negative coefficient, indicating that as the initial depression score increases, the association between the change in score and the proportion of dialogues reviewing the prior session’s recommendations decreases. Conversely, the main variable itself (i.e., review percentage), exhibited a positive coefficient, suggesting a correlation where a higher proportion of dialogues reviewing the previous session’s recommendations coincided to a greater change in score.

**Table 3:** Main Results from Generalized Estimating Equations modeling (with Interaction Terms) for the depression and anxiety datasets (note: therapist and organization fixed effects included but are not detailed due to presentation complexity)

Variable	PHQ9		GAD7	
	**Coefficient (*p*-value)**	**Coefficient CI [0.025 0.975]**	**Coefficient (*p*-value)**	**Coefficient CI [0.025 0.975]**
Intercept	-1.93 (ns)	[-6.40, 2.55]	-2.07 (ns)	[-5.56, 1.41]
**Log (Review Percentage)**	0.61 (0.032*)	[0.05, 1.17]	0.23 (ns)	[-0.11, 0.56]
Number of Dialogues/Session	0.04 (0.007*)	[0.01, 0.07]	0.01 (ns)	[-0.01, 0.04]
Number of Action Recommendations/Previous Session	0.01 (ns)	[-0.05, 0.07]	0.03 (ns)	[-0.04, 0.09]
Client's Initial Score	0.09 (0.006*)	[0.03, 0.015]	0.16 (0.000*)	[0.09, 0.22]
Days Examined	0.02 (ns)	[-0.06, 0.11]	0.02 (ns)	[-0.04, 0.08]
Log (Review Percentage): Initial Score	-0.02 (.045*)	[-0.04, -0.00]	-0.02 (ns)	[-0.05, 0.004]
Log (Review Percentage): Number of Dialogues/Session	-0.01 (ns)	[-0.03, 0.01]	NA	NA

^*^ denotes *p* < 0.05, "ns" denotes lack of statistical significance, “NA” denotes non-applicable. Variables transformed: review percentage logged, score change cube-rooted (the dependent variable). All significant associations were verified via bootstrap resampling (1000 samples)

This finding prompted us to employ a further exploratory analysis, in which the GEE analyses were performed again separately for each initial category of depression (PHQ-9) and anxiety (GAD-7), but this time without the interaction term. The categories for PHQ-9 are mild (5–9), moderate (10–14), moderately-severe (15–19), and severe (20–27) depression; and for GAD-7, mild (5–9), moderate (10–14), and severe (15–21) anxiety. The significance of the initial depression/anxiety score was nullified in these analyses (*p*-values ranged between 0.073 and 0.970), thereby reducing its impact across both datasets. However, within the PHQ-9 dataset, for the mild depression category (5–9), the review percentage was found to be significantly associated with the change in score (*p* = 0.024, Table 4), but not for other baseline depression categories (*p*-values ranged between 0.284 and 0.76, Table 4). For GAD-7, for all categories of initial score, no statistically significant associations of the review percentage were found (*p*-values ranged between 0.432 and 0.666).

**Table 4:** Main Results from Generalized Estimating Equations modeling for the depression dataset, partitioned by baseline depression categories (note: therapist and organization fixed effects included but are not detailed due to presentation complexity)

Variable	Baseline depression category	PHQ-9 Coefficient (*p*-value)	Coefficient CI [0.025, 0.975]
Intercept	Mild	2.56 (ns)	[-4.18, 9.30]
	Moderate	-14.07 (0.007*)	[-24.36, -3.78]
	Moderately severe	21.52 (0.001*)	[8.52, 34.53]
	Severe	6.30 (0.004*)	[1.99, 10.62]
**Log (Review Percentage)**	Mild	0.22 (0.024*)	[0.03, 0.42]
	Moderate	-0.13 (ns)	[-0.37, 0.11]
	Moderately severe	0.03 (ns)	[-0.14, 0.19]
	Severe	-0.05 (ns)	[-0.17, 0.06]
Number of Dialogues/Session	Mild	0.07 (0.002*)	[0.03, 0.11]
	Moderate	0.05 (0.027*)	[0.01, 0.09]
	Moderately severe	-0.04 (0.010*)	[-0.07, -0.01]
	Severe	-0.01 (ns)	[-0.03, 0.02]
Number of Action Recommendations/Previous Session	Mild	-0.04 (ns)	[-0.16, 0.07]
	Moderate	-0.01 (ns)	[-0.07, 0.05]
	Moderately severe	0.06 (ns)	[-0.00, 0.12]
	Severe	-0.07 (ns)	[-0.14, 0.00]
Client's Initial Score	Mild	-0.40 (ns)	[-0.85, 0.04]
	Moderate	0.06 (ns)	[-0.39, 0.51]
	Moderately severe	-0.42 (ns)	[-1.00, 0.16]
	Severe	-0.13 (ns)	[-0.38, 0.13]
Days Examined	Mild	-0.03 (ns)	[-0.14, 0.09]
	Moderate	0.25 (0.002*)	[0.09, 0.40]
	Moderately severe	-0.25 (0.010*)	[-0.44, -0.06]
	Severe	-0.04 (ns)	[-0.14, 0.05]

^*^ denotes *p* < 0.05, "ns" denotes lack of statistical significance. Variables transformed: review percentage logged, score change cube-rooted (dependent variable). All significant associations were verified via bootstrap resampling (1000 samples)

**Fig. 2:**
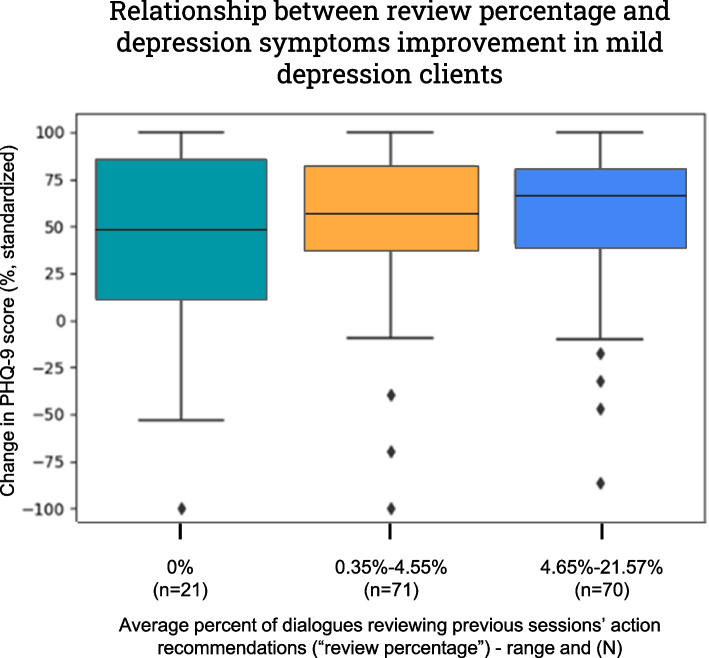
Relationship between review percentage and reduction of depression symptoms in mild depression baseline clients (box plot)

For visualization of this finding, we divided the initial mild depression clients (*n* = 162) into 3 groups: those who on average, across all of their analyzed therapeutic sessions, did not have any dialogues reviewing previous session action recommendations (i.e., average review percentage of 0%, *n* = 21), and two other groups roughly equal in sample size (*n* = 71, average review percentage range of 0.35%-4.55%, and *n* = 70, average review percentage range of 4.65%-21.57%), using the standardized change in depression score (Fig. 2). This visualization shows a trend of increasing change in score (i.e., increased improvement in depression symptoms) as the range of review percentage increases.

## Discussion

The current study explored the correlation between therapists' activation recommendations and their subsequent review, and changes in clients' symptoms of depression and anxiety within predominantly time-limited behavioral health interventions. Utilizing advanced language models and classification algorithms, we analyzed a large dataset of therapy sessions. Findings suggested that therapists providing behavioral therapy in community-based programs recommended during *each session* between 1 to 8 activities to engage in outside of therapy, with an average of 4.7 activity recommendations per session. However, only half of the sessions included a review of past action recommendations. Results further indicated that reviewing the previous session's action recommendations was associated with greater changes in depression scores for clients with mild depression. Review of past action recommendations was not associated with change in depressive symptoms scores for individuals with baseline moderate or severe depression or with change in anxiety symptoms.

The current study’s focus on activation recommendations aimed to improve understanding of the practical challenges that therapists encounter in implementing EBPs consistently. This allowed us to gain a more nuanced understanding of therapists' practices and the potential barriers they encounter when implementing evidence-informed intervention strategies. The behavioral health organizations studied in this trial mostly offered time-limited therapy. In this context, a correlation emerges between symptom improvement for clients with mild depressive symptoms and an active therapeutic approach that emphasizes behavioral activation throughout the treatment. The art of therapy involves a delicate balance between active listening, validation, and empathy toward clients' concerns, while also adopting a proactive stance that focuses on activation recommendations. The findings suggest that for therapists who tend to recommend multiple actions outside the session, engaging clients in the review and implementation of action recommendations from previous sessions could potentially lead to improved treatment outcomes. This cautiously underscores a potential benefit from integration of active and goal-oriented elements into therapy, possibly leading to improved outcomes for individuals with mild depression [11].

The complexity of these findings merits a highly important emphasis: this study is fundamentally observational, and making causal claims is challenging. The observed associations could be interpreted in various ways. While it is plausible that the review of activation recommendations led to improved depression outcomes, it is equally possible that therapists of clients who showed improvements were more likely to review activation recommendations (i.e., reversed causality). Alternatively, therapists might find it easier to review activation recommendations when clients are improving, and might shift strategies when progress is not evident, effectively focusing on new information instead of revisiting unproductive content. Therefore, the directionality of the associations found in this investigation remains unclear. We acknowledge this as an elemental limitation of the current study and emphasize that further research is needed to disentangle these potential influences and gain a more comprehensive understanding of the dynamics at play in therapeutic interactions. However, even if we assume the directionality of these associations is that greater review leads to improved depression outcomes, the lack of association between review of action recommendations in moderate to severe levels of depression is surprising, considering previous research findings that suggest prescribing clients with between-session activities is predictive of better outcomes [10].

Findings imply therapists intend to help clients make meaningful changes by providing at least 4 recommendations about actions to engage in outside of therapy; however, overwhelming workloads, administrative burden, and therapist drift may create a potential challenge in maintaining consistent follow-up and accountability [21, 38]. Additionally, there are several possible explanations for why this particular activation recommendation and review showed positive correlation only in mild depression—but not in more moderate or severe depressive symptoms—and why it did not exhibit any correlation for anxiety. A likely explanation for these findings could be the presence of confounding variables. For instance, clients with mild depression may engage with their therapists in ways that promote greater activation recommendations and review compared to clients with anxiety or more severe depressive symptoms, due to the differential treatment response based on the severity of depression. Further, clients with mild depression may have greater adaptive coping skills, rendering them more responsive to following their therapist’s guidance beyond sessions [39]. For instance, they might engage in more frequent pleasurable activities or benefit emotionally from such participation to a greater extent. Conversely, individuals with moderate or severe depression may require a more comprehensive and standardized behavioral activation intervention that addresses their specific needs beyond activation recommendation. The absence of similar correlations in clients with anxiety may indicate that anxiety symptoms necessitate distinct therapeutic strategies other than behavioral activation [40], highlighting the heterogeneity of mental health conditions and the importance of tailored interventions.

An examination of the descriptive results reveals that approximately half of the sessions included a review of the past session’s assigned activation recommendation. This suggests that therapists may not have consistently presented homework or tested it in a systematic manner. This finding aligns with a recent systematic review conducted by Ryum, Bennion, and Kazantzis [12], which indicates that certain therapist behaviors can support clients in establishing realistic and clear expectations about homework, fostering engagement, and promoting symptom improvement. Specifically, collaborative activities such as designing, planning, and reviewing homework in line with clients' goals and values; aligning the homework with session takeaways; providing a comprehensive explanation and persuasive rationale for the homework; addressing potential challenges and barriers to task engagement; offering a written summary of the homework; considering and incorporating client feedback; and being responsive to clients’ evolving needs and circumstances are among the therapist behaviors that appear to be significant factors in facilitating positive outcomes [12].

There are likely additional potential factors influencing the therapist's engagement in the review of action recommendations and their subsequent impact on the reduction of symptoms, primarily in clients presenting with mild depression. Various elements might underpin this relationship, operating through distinct pathways. For instance, the frequency of reviewing action recommendations may be influenced by the quality of the collaborative therapeutic relationship [7]. Therapists who are better trained, exhibit enhanced active listening skills, or foster a strong client involvement in therapy may be more inclined to engage in the meticulous review of previously assigned actions. Unraveling these correlates promises a deeper understanding of the interplay between therapeutic dynamics and client outcomes.

This study has some limitations that warrant consideration. First, the data were derived from a diverse but specific set of therapy programs that might have different important respects from other interventions and programs, potentially limiting the generalizability of the findings to other treatment settings [21, 41]. Additionally, although efforts were made to control for confounding factors, the observational design of the study may still leave room for unmeasured variables to influence the results. Moreover, the study focused specifically on time-limited therapy, and thus, the findings may not apply to longer-term therapies or different treatment modalities. Calculating the exact magnitude of the correlation was difficult due to data transformations for skewness and outliers. Although the correlation coefficient shows a significant link between review percentage and depression score changes in mild depression individuals, an examination of Fig. 2 indicates that the actual effect size seems to be relatively small. Additionally, this study did not assess the relative importance therapists assign to their recommendations or the clients' perceptions of these recommendations. A subsequent investigation is warranted to explore the significance of reviewing these recommendations, offering insights into their nature, potential behavioral connections, and the relevance of reviewing them in subsequent sessions. Additionally, while the current study focused on the immediate follow-up of action recommendations within consecutive therapy sessions, a broader exploration of their topic consistency as a potential predictor of outcomes throughout the entire course of treatment opens a promising avenue for future research in understanding the dynamic nature of therapeutic discussions and their impact on overall treatment effectiveness. Furthermore, the utilization of self-report measures for depression and anxiety introduces the possibility of response biases and the inherent subjectivity associated with these assessments. Future trials should also aim to investigate potential moderators for homework review as emphasized in clinical research and practice guidelines [42]. Lastly, the associations identified in this study, although statistically significant, emerged through post-hoc analyses and may be different under multiple comparisons correction. Therefore, results should be interpreted cautiously and regarded as preliminary insights, necessitating further validation in subsequent studies.

## Conclusion

In conclusion, this study expands the understanding of the relationship between reviewing action recommendations within therapy sessions and improvements in depression symptoms for clients with mild depression. The study additionally demonstrates the feasibility of measuring these therapeutic ingredients in usual care. The findings underscore the possible importance of integrating active therapeutic elements that promote behavioral activation in the treatment of mild depression. Supervision, particularly that which is data-driven, could play a crucial role in addressing therapist drift and promoting adherence to treatment protocols [43]. Nonetheless, it is essential to acknowledge the limitations of this study and the need for further research to explore differential treatment responses based on depression severity, causality, and the generalizability of these findings to other clinical populations. To advance the field, future research should focus on real-world data derived from diverse treatment settings, rely on session data rather than self-report measures, and investigate a broader range of therapeutic interventions to inform evidence-based practice in clinical psychology.

### Clinical implications

This study focuses on the intervention strategies of assigning and reviewing action recommendations. The findings reveal a generally low follow-through with previously assigned action recommendations, indicating limited continuity between sessions. Moreover, findings suggest a potential link between follow-up practices and enhanced treatment outcomes for clients with mild depression, a relationship whose directionality warrants further investigation. Once confirmed, these insights can inform clinical practice and contribute to the refinement of evidence-based treatment protocols for depression.

## Data Availability

The data that support the findings of this study are not publicly available due to privacy and ethical restrictions.

## Abbreviations

*AI*: Artificial Intelligence
*BART*: Bidirectional Auto-Regressive Transformers
*EBP*: Evidence-based practice
*GAD-7*: Generalized Anxiety Disorder-7
*GEE*: Generalized Estimating Equations
*PHQ-9*: Patient Health Questionnaire-9
