# Correction: Action recommendations review in community-based therapy and depression and anxiety outcomes: a machine learning approach

**DOI:** 10.1186/s12888-024-05655-w

**Published:** 2024-03-13

**Authors:** Amit Spinrad, C. Barr Taylor, Josef I. Ruzek, Samuel Jefroykin, Tamar Friedlander, Israela Feleke, Hila Lev-Ari, Natalia Szapiro, Shiri Sadeh-Sharvit

**Affiliations:** 1 Eleos Health, 117 Kendrick Street, Suite 300, 02494 Needham, MA USA; 2 Center for m2Health, Palo Alto University, Palo Alto, CA USA; 3 Department of Psychiatry, Stanford Medical Center, Stanford, CA USA

Following publication of the original article [1], the authors identified an error in the author name of Amit Spinrad.

The incorrect author name is: Amit Spinard.

The correct author name is: Amit Spinrad.

The author group has been updated above and the original article [1] has been corrected.
